# Supplementary material for: MiR-92 Family Members Form a Cluster Required for Notochord Tubulogenesis in Urochordate Ciona savignyi
Source: Genes (Basel). 2021 Mar 12;12(3):406. doi: 10.3390/genes12030406 (PMC8001136; doi:10.3390/genes12030406)
Supplement: Supplementary file 1 [file genes-12-00406-s001.pdf]

Supplementary Materials:

Table S1. The sequence of the gRNAs for electroporation.

gRNA	The sequence of gRNA	Position
sgRNA-mm	CACCTGTCCCGGCCGATCTTGGG	reftig_1 DNA:reftig reftig:CSAV2.0:reftig_1:1:6668731:1 REF 1335597-1335619
sgRNA-Dicer	TAACAGTATTGCACCTGTCCCGG	reftig_1 DNA:reftig reftig:CSAV2.0:reftig_1:1:6668731:1 REF 1335586-1335608
sgRNA-Drosha	ATCAAGCGTATATAGCAGTCAGG	reftig_1 DNA:reftig reftig:CSAV2.0:reftig_1:1:6668731:1 REF 1335529-1335551
Control gRNA	GCTTTGCTACGATCTACATT	

Table S2. The distribution traits of miR-92 among different species.

Species	Name of miRNA	Gene Location	Gene Organization	+/− Strand
Ciona savignyi	csa-mir-92a	reftig_1: 1336385-1336487	Intergenic region	+
	csa-mir-92b	reftig_1: 1335375-1335474	Intergenic region	+
	csa-mir-92c	reftig_1: 1335536-1335626	Intergenic region	+
Ciona robusta	cin-mir-92a	3p: 1864426-1864526	Intergenic region	+
	cin-mir-92b	1p: 2635676-2635776	Intergenic region	−
	cin-mir-92c	3p: 1865219-1865319	Intergenic region	+
	cin-mir-92d	3p: 1864796-1864851	Intergenic region	+
	cin-mir-92e	1p: 2635396-2635447	Intergenic region	−
Homo sapiens	hsa-mir-92a-1	chr13: 91351314-91351391	Promoter region	+
	hsa-mir-92a-2	chrX: 134169538-134169612	Exon	−
	hsa-mir-92b	chr1: 155195177-155195272	Promoter region	+
	hsa-mir-17	chr13: 91350605-91350688	Promoter region	+
	hsa-mir-18a	chr13: 91350751-91350821	Promoter region	+
	hsa-mir-19a	chr13: 91350891-91350972	Promoter region	+
	hsa-mir-20a	chr13: 91351065-91351135	Promoter region	+
	hsa-mir-19b-1	chr13: 91351192-91351278	Promoter region	+
Mus musculus	mmu-mir-92a-1	chr14: 115044427-115044506	Promoter region	+
	mmu-mir-92a-2	chrX: 52741838-52741928	Intergenic region	−
	mmu-mir-92b	chr3: 89227116-89227198	Promoter region	−
	mmu-mir-17	chr14: 115043671-115043754	Promoter region	+
	mmu-mir-18a	chr14: 115043851-115043946	Promoter region	+
	mmu-mir-19a	chr14: 115044000-115044081	Promoter region	+
	mmu-mir-20a	chr14: 115044157-115044263	Promoter region	+
	mmu-mir-19b-1	chr14: 115044305-115044391	Promoter region	+
Drosophila pseudoobscura	dps-mir-92a	2: 23635276-23635369	unknown	−
	dps-mir-92b	2: 23630760-23630859	unknown	−
	dps-mir-92c	3: 8679866-8679980	unknown	+
Gallus gallus	gga-mir-92-1	chr1: 148023263-148023340	Intergenic region	−
	gga-mir-92-2	chr4: 3994560-3994640	Intergenic region	−
	gga-mir-17	chr1: 148023974-148024058	Intergenic region	−
	gga-mir-18a	chr1: 148023819-148023911	Intergenic region	−
	gga-mir-19a	chr1: 148023685-148023765	Intergenic region	−
	gga-mir-20a	chr1: 148023499-148023596	Intergenic region	−
	gga-mir-19b	chr1: 148023376-148023462	Intergenic region	−
Xenopus laevis	xla-mir-92a-1	chr2L: 107331786-107331863	unknown	−
	xla-mir-92a-2	chr8L: 48821825-48821900	unknown	−
	xla-mir-92a-3	chr8S: 74307587-74307654	unknown	−
	xla-mir-92a-4	chr2S: 91488294-91488353	unknown	−
	xla-mir-92b	Sc000103_chrNA: 303236-303296	unknown	+

	xla-mir-17-2	chr2L: 107332477-107332538	unknown	−
	xla-mir-18b	chr2L: 107332346-107332408	unknown	−
	xla-mir-19a	chr2L: 107332210-107332271	unknown	−
	xla-mir-20a	chr2L: 107332044-107332104	unknown	−
Danio rerio	dre-mir-92a-1	chr1: 3297367-3297451	Exon or intron	−
	dre-mir-92a-1	chr9: 53436280-53436374	Exon or intron	−
	dre-mir-92b	chr16: 42898429-42898510	Exon or intron	+
	dre-mir-17a-1	chr1: 3298112-3298249	Exon or intron	−
	dre-mir-18a	chr1: 3298006-3298088	Exon or intron	−
	dre-mir-19a	chr1: 3297848-3297936	Exon or intron	−
	dre-mir-20a	chr1: 3297603-3297755	Exon or intron	−
	dre-mir-19b	chr1: 3297484-3297570	Exon or intron	−
Branchiostoma floridae	bfl-mir-92a-1	GG666632.1: 777177-777277	unknown	−
	bfl-mir-92a-2	GG666632.1: 774437-774518	unknown	−
	bfl-mir-92a-3	GG666632.1: 776303-776384	unknown	−
	bfl-mir-92b	GG666522.1: 372710-372810	unknown	+
	bfl-mir-92c	GG666522.1: 372242-372441	unknown	+
	bfl-mir-92d	GG666581.1: 210790-210872	unknown	+
	bfl-mir-19	GG666583.1: 3476282-3476363	unknown	−
Saccoglossus kowalevskii	sko-mir-92a	GL015959.1: 55312-55412	unknown	−
	sko-mir-92b	GL015959.1: 42157-42257	unknown	−
	sko-mir-92c	GL015959.1: 40764-40864	unknown	−
Capitella teleta	cte-mir-92a	KB294417.1: 594329-594429	unknown	−
	cte-mir-92b	KB294417.1: 595219-595319	unknown	−
	cte-mir-92c	KB294417.1: 594205-594305	unknown	−
Strongylocentrotus purpuratus	spu-mir-92a	KN917153.1: 419786-419885	unknown	−
	spu-mir-92b	KN917153.1: 421925-422025	unknown	−
	spu-mir-92c	KN917153.1: 420245-420345	unknown	−
	spu-mir-92d	AAGJ05134155.1: 22230-22288	unknown	+
	spu-mir-92e	AAGJ05134155.1: 22757-22817	unknown	+

Table S3. The number and percentage of the csa-miR-92c-KO embryos.

	**Eggs for Electroporation**	**Developmental Embryos**	**Cas9-Positive Embryos (C)**	**Embryos with Phenotype (P)**	**P/C**
16 hpf (sgRNAs)	200–300	25	15	6	40%
16 hpf (sgRNA-control)	200–300	27	21	3	14.3%
21 hpf (sgRNAs)	200–300	21	13	4	30.8%
21 hpf (sgRNA-control)	200–300	33	25	4	16%
31 hpf (sgRNAs)	200–300	32	21	2	9.5%
31 hpf (sgRNA-control)	200-300	31	24	3	12.5%

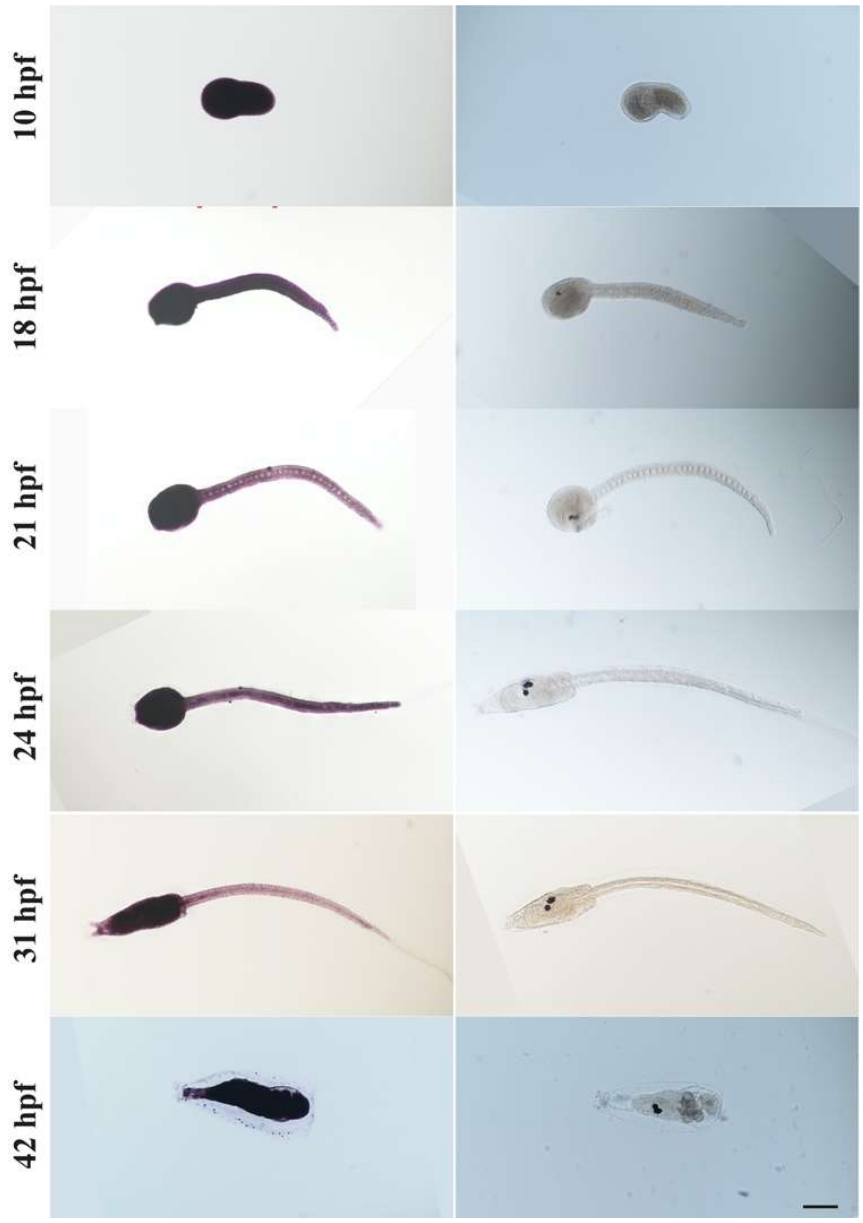

Figure S1. Expression patterns of csa-miR-92c detected by *in situ* hybridization. Embryos and larvae at 10, 18, 21, 24, 31, and 42 hpf were hybridized with LNA probes of csa-miR-92c. The developmental stages were indicated in the left. The results of sense probes were indicated in the right. The signals of csa-miR-92c were detected in the whole body at 10 hpf and 42 hpf and were found to be expressed in the trunk, notochord cells, and epithelial cells at 18, 21, 24, and 31 hpf. The red frame indicated the regions of zoom-in images in the middle column. Scale bars represent 100 μm.

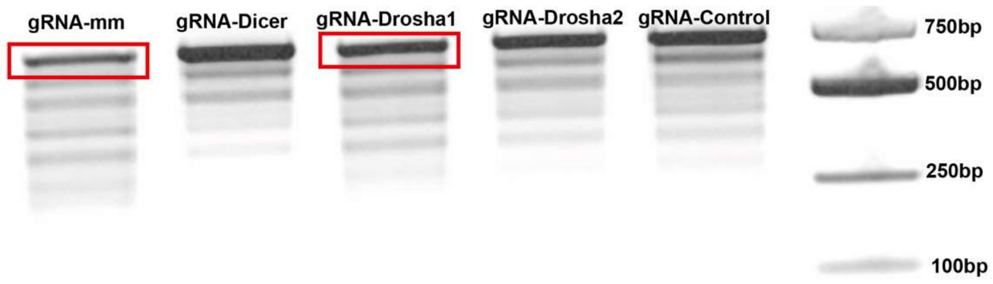

Figure S2. The sgRNA efficiency verification by T7 endonuclease I assay. The efficiency of four gRNAs in *Ciona* was detected by T7 endonuclease I assay. The percentages of gene modification by gRNA-mm and gRNA-Drosha1 (pointed in the red box) were 11.3% and 1.4%, respectively. gRNA-mm: the gRNA designed at the mature sequence of csa-miR-92c; gRNA-Dicer: the gRNA designed at the active site of Dicer of csa-miR-92c; gRNA-Drosha1: the gRNA designed at the active site of Drosha of csa-miR-92c; gRNA-Drosha2: the gRNA designed at the active site of Drosha of csa-miR-92c; gRNA-control, and marker.

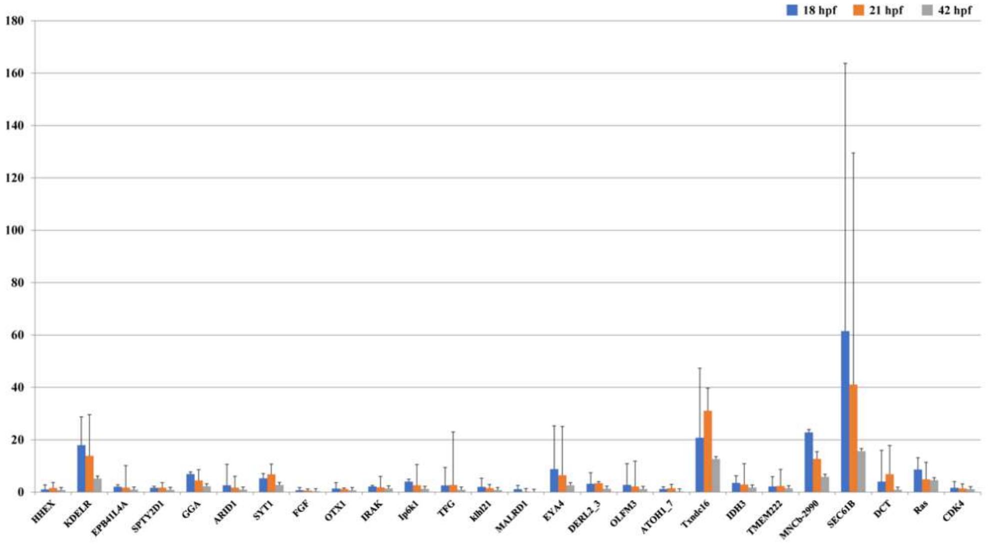

Figure S3. The relative expression levels of the downregulated target genes of csa-miR-92c. The relative expressions of these targeted genes were downregulated from 18 hpf to 42 hpf.

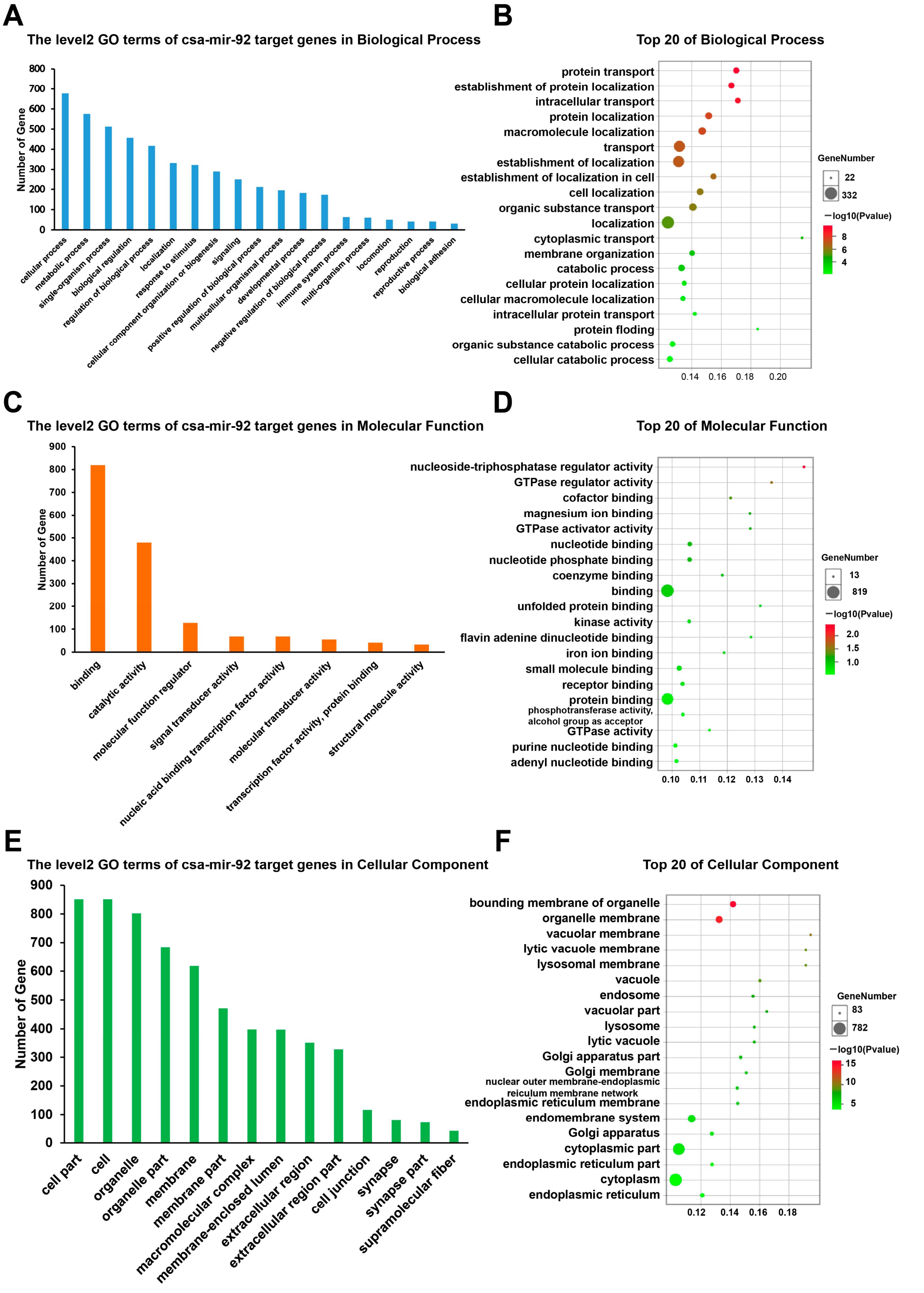

Figure S4. Gene ontology analysis of putative target genes of csa-miR-92. (**A**,**C**,**E**) The level2 Go terms of csa-mir-92 target genes in Biological process (**A**), molecular function (**C**), and cellular component (**E**) of Gene Ontology. The abscissa represents the more detailed first-level classification of the three ontologies of GO. The ordinate represented the number of genes contained in each category entry. The top 20 of Biological process (**B**), molecular function (**D**), and cellular component (**F**) of Gene Ontology. The left side of the bubble chart was the name of biological process, molecular function, or cellular component, and the below of the bubble chart was the rich factor. The bubble size indicated the gene number. The color bar depicted the level of the −log10 (*p*-value). Green or red on the bubble indicated that the level of the −log10 (*p*-value) is decrease or increase, respectively.

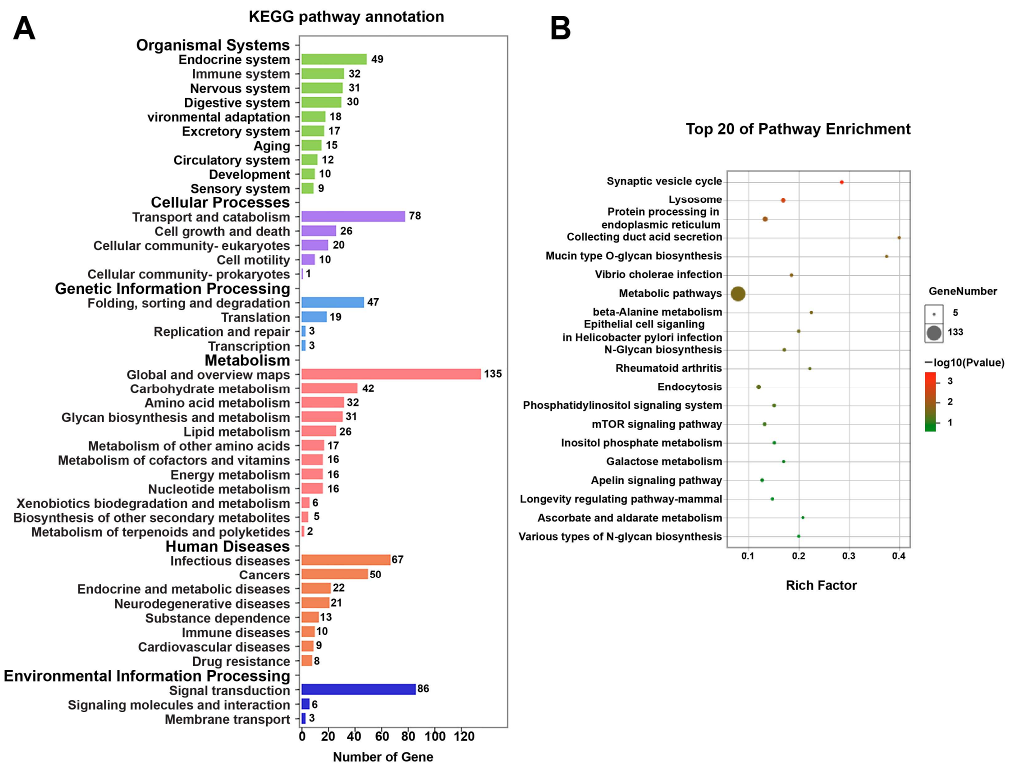

Figure S5. Pathway enrichment analysis of putative target gene of csa-mir-92. (**A**) The KEGG pathway annotation. The information on the left of the chart was divided into six categories, namely Organismal Systems, Cellular Processes, Genetic information Processing, Metabolism, Human diseases, and Environmental Information Processing, respectively. Different colors represented the different categories. The number of genes was marked on the right of the bar. (**B**) The top 20 pathway enrichments of the KEGG analysis. The left side of the bubble chart was the information of pathway, and the below of the bubble chart was the rich factor. The bubble size indicated the gene number. The color bar depicted the level of the −log10 (*p*-value). Green or red on the bubble indicated the level of the −log10 (*p*-value) was decreased or increased, respectively.
